# Freely accessible software for recruitment prediction and recruitment monitoring of clinical trials: A systematic review

**DOI:** 10.1016/j.conctc.2024.101298

**Published:** 2024-04-22

**Authors:** Philip Heesen, Malgorzata Roos

**Affiliations:** a Faculty of Medicine, University of Zurich, Raemistrasse 71, 8006, Zurich, Switzerland; b Department of Biostatistics at the Epidemiology, Biostatistics and Prevention Institute, University of Zurich, Hirschengraben 84, 8001, Zurich, Switzerland

**Keywords:** Clinical trial, Design stage, Free-of-charge open-source software, Planning, Recruitment prediction, Systematic review

## Abstract

**Background:**

The successful completion of clinical trials ultimately depends on realistic recruitment predictions. Statistical methods for recruitment prediction implemented in a free-of-charge open-source software could be routinely used by researchers worldwide to design clinical trials. However, the availability of such software implementations is currently unclear.

**Methods:**

Two independent reviewers conducted a systematic review following PRISMA guidelines. Eligible articles included English publications focused on statistical methods for recruitment prediction and monitoring that referred to software implementations. The list of articles retrieved from well-established data bases was enriched by backtracking of references provided by eligible articles. The current software availability and open-source status were tabulated.

**Results:**

We found 21 eligible articles, 7 of which (33 %) provide freely accessible software. Ultimately, only one article provides a link to an easy-to-comprehend, well-documented, and currently directly applicable free-of-charge open-source software. The lack of availability is mainly caused by blocked access and outdated links.

**Conclusions:**

While several software implementations exist for recruitment prediction, only a small fraction is freely accessible. These results highlight the need for future efforts to achieve free access to well-documented software implementations supporting researchers in routinely using statistical methods to arrive at realistic recruitment predictions in clinical trials.

## Highlights

Key findings

• Statistical methods for recruitment modeling have been developing since the 1980s.
• Free-of-charge open-source software implementations are needed worldwide.
• Only few articles offer such software for recruitment prediction of clinical trials.

What this adds to what is known?

• The lack of free access to software makes the use of statistical methods difficult.
• Links to free-of-charge open-source software promote the use of statistical methods.
• Articles listed here help navigate the overwhelming number of statistical methods.

What is the implication and what should change now?

• Freely accessible software would support realistic recruitment predictions.
• Future authors should explicitly mention their open-source software in articles.
• They should also provide user-friendly documentation and instructive examples.

## 1 Background

High-quality clinical trials (CTs) that have recruited an adequate number of participants reliably discover health-promoting therapies worldwide. The successful completion of a CT ultimately depends on both accurate recruitment prediction and accurate recruitment monitoring. This also applies to randomized controlled trials, an integral part of CTs. Although there is commercial software for recruitment modeling, its cost is prohibitive for many research groups particularly, though not exclusively, in low- and middle-income countries [1]. This lack of free-of-charge specialized software to assist realistic decision-making can impede the successful completion of CTs [2], entirely wasting limited resources [3].

Recruitment prediction is extremely challenging. For example, researchers must consider different options to balance both recruitment rates and the time needed to recruit enough patients. Statistical methods for recruitment modeling have been developing since the 1980s [[4], [5], [6]], and an overwhelming number of relevant statistical methods has accumulated over the years [2,[7], [8], [9], [10]]. Ideally, such statistical methods for recruitment modeling should be implemented in a free-of-charge open-source software [11] so that principal investigators and funders can routinely use these methods to predict and monitor recruitment rates, counts of subjects accrued, and recruitment duration [2].

Unfortunately, only 1 % of researchers uses simulations based on statistical methods to support pretrial planning and only 10 % use statistical models to predict recruitment at the design stage [3]. Gkioni et al. [3] speculate that this undesirable situation is caused by the considerable time and training required by researchers to implement complex statistical models themselves. However, this study poses another hypothesis: such findings could be the result of a lack of real free-of-charge open-source software implementations of complex statistical methods for recruitment prediction and monitoring. To evaluate this hypothesis, we clarify the current availability of such software in this comprehensive systematic review.

## 2 Methods

To clarify current software availability, we conducted a transparent systematic review closely following Aromataris [12] and PRISMA guidelines [13] focused on English articles dealing with statistical methods for recruitment modeling that implemented software, provided a link to the software, or provided a file containing software as supplementary material. Only full papers (i.e., no abstracts) were included.

To detect relevant articles, two independent reviewers conducted a systematic literature review based on three supplementary steps. In the first step, we used a web search engine (PubMed, Google Scholar, Embase, Web of Science) with the search string (“recruitment” OR “accrual” OR “enrollment”) AND (“trial” OR “RCT” OR “CT” OR “clinical trial” OR “study”) AND (“software” OR “code” OR “R” OR “open source”). This search string was capable of detecting the benchmark studies [[14], [15], [16], [17]]. On May 3, 2023, this search string was extended by adding AND (“planning” OR “plan” OR “protocol”) and executed on May 14, 2023. Literature records were deduplicated with EndNote. In the second step, we tracked down eligible articles compiled by the web search in the first step and extended the list of eligible articles by systematically backtracking the references mentioned in these publications. Finally, we screened the content of packages listed on CRAN Task View: Clinical Trial Design, Monitoring, and Analysis [18] to track down relevant software and additional articles.

Initially, both reviewers reached a consensus on which outcome categories to extract and established a unified and standardized coding of variables for data collection. The outcome categories included the current free access to the software (no, yes) and its open-source status (yes = "free-of-charge open-source software”, request = "code is available on request from the authors of the article”, closed = "closed-source software”). In addition, statistical methods were categorized (F = "Frequentist”, B = "Bayes”, EB = "Empirical Bayes”) and their level of intricacy was assigned (low, medium, high). “Frequentist” statistical methods use only data for statistical analysis, “Bayes” methods combine data with prior distributions elicited from experts, and “Empirical Bayes” use the same Bayesian framework as Bayes methods but reuse data to estimate the parameters of prior distributions. The level of intricacy by no means reflects the quality, user-friendliness, and performance of the software but rather our subjective perception of the workload necessary to comprehend and apply statistical methods implemented in the software. Both reviewers independently tested the availability and usability of the software referenced in the articles included in the systematic review. Discrepancies between reviewers were resolved by discussions. Subsequently, the properties of eligible articles were tabulated with respect to outcome categories and summarized using absolute and relative frequencies. To assess the confidence, the DescTools package in R was used to compute Wilson 95 % confidence intervals (95%CI). All results are summarized in Table 1, including comments relevant to readers and links to software.

**Table 1:** Characteristics of articles found by the systematic review tabulated according to their free access (no, yes), open-source (yes, request, closed), statistical methods (F = "Frequentist”, B = "Bayes”, EB = "Empirical Bayes”), and intricacy (low, medium, high). Software implementations to which we have access are listed first. The remaining implementations are ordered according to their open-source status (yes, request, closed). Each of these groups is organized according to the publication date. The number in brackets corresponds to the citation number used in this paper.

Article	Access	Open-source	Methods	Intricacy	Comments
Zhang et al. (2012) [19]	yes	yes	B	high	Code in Supporting Information
Jiang et al. (2016) [20]	yes	yes	B	medium	Archived on 2023-08-19: https://cran.r-project.org/src/contrib/Archive/accrual/
Spies et al. (2021) [1]	yes	yes	F	low	Software on GitHub: https://github.com/spiesruan/TrialRecruitmentTool
Urbas et al. (2022) [17]	yes	yes	B	high	Software on GitHub: https://github.com/SzymonUrbas/ct-recuitment-prediction
Mountain et al. (2022) [21]	yes	yes	EB	high	Code in Supporting Information
Bütikofer et al. (2022) [22]	yes	yes	F	low	Software: https://CRAN.R-project.org/package=accrualPlot
Perperoglou et al. (2023) [23]	yes	yes	B	high	Code in Supporting Information
Bagiella et al. (2001) [24]	no	yes	B, F	medium	Obsolete link to software
Carter (2004) [14]	no	yes	F	low	Obsolete link to software
Carter et al. (2005) [25]	no	yes	F	low	Obsolete link to software
Ying et al. (2008) [26]	no	yes	B	medium	Obsolete link to software
Ying et al. (2013) [27]	no	yes	B, F	medium	Obsolete link to software
Moussa (1984) [28]	no	request	F	low	Implementation of methods developed by Lee (1983) [5]
Gajewski et al. (2008) [29]	no	request	B	medium	Jiang et al. (2016) [20] use these methods
Heitjan et al. (2015) [9]	no	request	B, F	medium	
Lan et al. (2019) [30]	no	request	B	high	
Abbas et al. (2007) [31]	no	closed	F	low	NA
Anisimov (2009) [32]	no	closed	EB	medium	NA
Anisimov (2009) [33]	no	closed	EB	medium	NA
Anisimov (2011) [15]	no	closed	EB	medium	NA
Liu et al. (2019) [16]	no	closed	B	medium	NA

## 3 Results

**Fig. 1:**
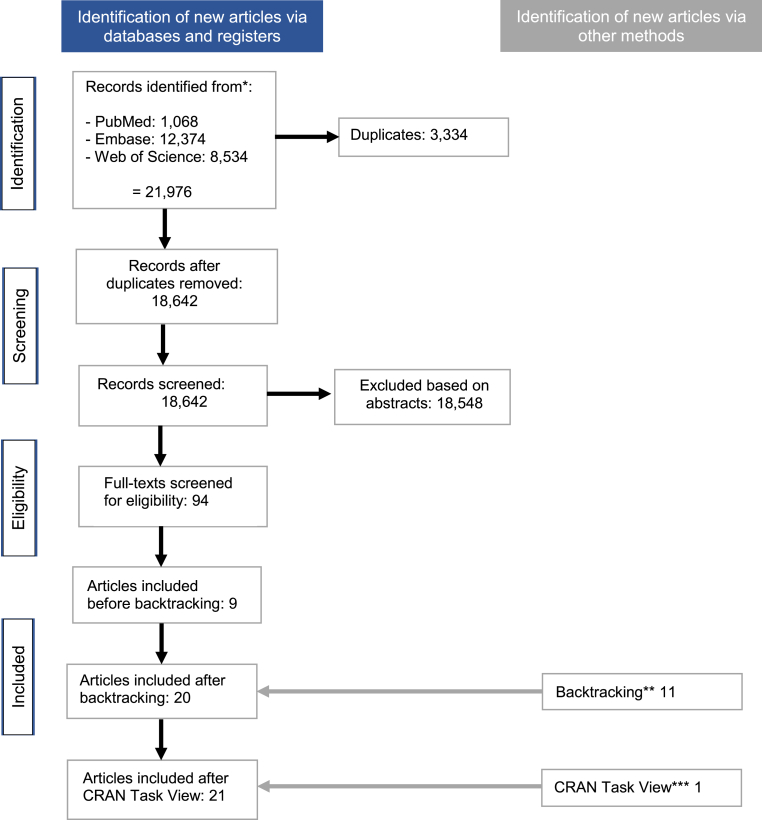
PRISMA flow-chart of the systematic review clarifying the availability of free-of-charge open-source software for recruitment prediction and monitoring of CTs.

While the PRISMA chart in Fig. 1 outlines the systematic review, Table 1 summarizes the characteristics of 21 eligible articles. Seven out of 21 articles (33 %, 95%CI(17 %, 55 %)) provide access to free-of-charge, open-source software that is directly applicable. The remaining 14 articles (67 %, 95%CI(45 %, 83 %)) refer to software that is not directly applicable for one of the following reasons: either an obsolete link to the software is provided 36 % (5/14, 95%CI(16 %, 62 %)), or an interested reader must submit a request to the authors to get access to the software 29 % (4/14, 95%CI(11 %, 55 %)), or a closed-source software is used 36 % (5/14, 95%CI(16 %, 62 %)).

The 7 articles that provide access to free-of-charge, open-source software have additional characteristics that impact their usability in practice. Four articles [17,19,21,23] deal with intricate statistical methods that require thorough statistical training necessary to use them. In contrast, the remaining 3 [1,20,22] articles are based on statistical methods that are easier to comprehend and use. However, the accrual package used by Jiang et al. [20] was archived on August 19, 2023, and is no longer freely accessible on CRAN. Moreover, the accrualPlot package [22] provided on CRAN is not accompanied by any article that specifies the statistical methods implemented in this package. For these reasons, only one easy-to-comprehend, well-documented, free-of-charge, open-source software provided by Spies et al. [1] on GitHub can currently be directly used for recruitment prediction and recruitment monitoring of clinical trials.

The interactive trial recruitment tool developed by Spies et al. [1] predicts trial recruitment duration. To compute confidence intervals of the trial recruitment duration predictions, the authors use the pragmatic simulation approach proposed by Carter [14] and implement Monte Carlo simulations of the Poisson process with constant recruitment rates for each site.

The complete trial recruitment tool provides interactive dashboards for planning and monitoring. The planning dashboard comprises single site and multisite planning. For single site planning, the input requires the specification of three parameters: the expected recruitment rate, the recruitment goal, and the level of confidence. The output provides the confidence interval of the trial recruitment duration prediction. For multisite planning, each site involved requires similar specifications to those for the single site. Moreover, initiation delays of sites can be easily incorporated. The output of the multisite planning provides the confidence interval of the whole multisite trial recruitment duration prediction. The monitoring dashboard comprises baseline parameters, a trial overview, and a site-specific information. If data from an ongoing trial are provided, the monitoring dashboard reports confidence intervals for the recruitment duration prediction that are based on the ongoing trial.

To explore the functionality of interactive dashboards developed by Spies et al. [1] hit the link https://github.com/spiesruan/TrialRecruitmentTool to GitHub and follow instructions. The preliminary step includes installation of R, RStudio and R libraries. The easiest way to use the interactive dashboard is to download the app.R file from GitHub, save it locally on the computer, open this file in RStudio and click on Run App button in RStudio.

## 4 Discussion

This comprehensive systematic review confirmed that real software implementations of complex statistical methods for recruitment prediction and monitoring are currently practically unavailable to wider audiences in the form of free-of-charge open-source software. In our opinion, this poor access is surprising, considering that the area of statistical methods for recruitment prediction and monitoring has been evolving since the 1980s. This lack of availability can hinder principal investigators and funders from routinely using statistical methods to predict and monitor recruitment rates, counts of subjects accrued, and recruitment duration, thus leading to the undesirable situation reported by Gkioni et al. [3].

In addition to this main contribution, our comprehensive systematic review of software implementations also indirectly offers a historical overview of statistical methods for recruitment prediction and recruitment monitoring, helping the principal investigators and funders to accurately navigate the numerous methods that have accumulated since 1980s. Note, however, that the methods implemented in the software listed in Table 1 do not include all existing statistical methods, but rather reflect the willingness of researchers to share the software with others. Indeed, there are more statistical methods for recruitment prediction and monitoring that have been reviewed elsewhere [2,[7], [8], [9], [10]].

In the review process, we encountered several obstacles. First, we found more eligible articles by backtracking the references of suitable publications rather than through the original systematic literature search. This clearly indicates that any mention of software implementation is frequently buried in the text rather than explicitly mentioned in the title, abstract, and keywords. Ultimately, our systematic review would have benefitted from a larger number of reviewers manually backtracking the citations of eligible articles. Because it is still possible that our literature review missed some links to free-of-charge open-source software, we would value feedback from readers who are aware of further eligible articles. Nevertheless, this systematic literature review considerably extends the two short software listings provided earlier [2,9] and provides a comprehensive up-to-date outline of software availability.

A second difficulty was that this systematic review also found articles with links to free-of-charge open-source software that were invalid. Thus, the original software can no longer be used, making the results reported in these articles computationally non-reproducible. Moreover, it revealed articles that contain methods that are highly relevant for recruitment prediction and monitoring, but these are not currently freely accessible. For example, Anisimov [32] and Liu et al. [16] mention the impact and benefits of their software implementations, which are in fact closed-access.

Authors and journals could take various actions to resolve the issue if the link was correct when published but later became outdated. The spectrum of these actions is very broad and ranges from moving the entire software implementation from an inactive to an active website to reimplementing the entire method in new software. Additionally, authors who reported on closed-access software may reconsider their decision and provide open access to their software implementation. The effective action depends on the real situation and the willingness of journals and authors to deal with these issues.

This systematic review provides immediate answers regarding the availability of software, but it also establishes an important objective basis for further efforts to achieve full free access to software implementations. Future authors could support this initiative by explicitly mentioning software implementations in abstracts and keywords and by providing software implementations online on Zenodo, OSF, GitHub or GitLab, thus supporting the contemporary initiative for open and reproducible research [11]. Moreover, they should provide user-friendly documentation and instructive examples of how to use the statistical methods implemented in their software in applications. In the long term, it could become standard to use well-documented, free-of-charge, open-source software to obtain insightful predictions of recruitment, supporting realistic decisions at the design stage of CTs and randomized controlled trials. Such realistic decisions would increase the chance that adequate numbers of participants are recruited and CTs are successfully completed, thus optimizing the use of limited resources and accelerating the discovery of health-promoting therapies worldwide.

## 5 Conclusions

The lack of specialized free-of-charge open-source software to assist recruitment prediction, recruitment monitoring, and realistic decision-making can impede the successful completion of clinical trials. Although statistical methods for recruitment modeling have been developing since the 1980s and an overwhelming number of relevant statistical methods have accumulated over the years, this systematic review reveals that a surprisingly low number of free-of-charge open-source software implementations can be directly used for the recruitment prediction and recruitment monitoring. Only one out of the 21 articles found here provides a link to an easy-to-comprehend, well-documented, and currently directly applicable free-of-charge open-source software. This software follows Carter [14] and implements Monte Carlo simulations of the Poisson process. Remaining statistical methods are currently not freely accessible to a wider audience. To improve access to software implementations and support the contemporary initiative for open and reproducible research, future authors should explicitly mention their open-source software implementations in abstracts, keywords, and articles and provide user-friendly documentation and instructive examples of how to use the statistical methods implemented in their software.

## Competing interests’ statement

The authors of this manuscript do not have any potential or perceived conflicts of interest to declare.

## Funding

This research did not receive any specific grant from funding agencies in the public, commercial, or not-for-profit sectors.

## Data availability

No data were used for the research described in the article.

## Ethics approval

Not required.

## Consent for publication

Not applicable.

## CRediT authorship contribution statement

**Philip Heesen:** Writing – review & editing, Visualization, Methodology, Investigation, Formal analysis, Conceptualization. **Malgorzata Roos:** Writing – review & editing, Writing – original draft, Supervision, Methodology, Investigation, Formal analysis, Conceptualization.

## Declaration of generative AI and AI-assisted technologies in the writing process

During the preparation of this work the author(s) did not use any generative AI and AI-assisted technologies.

## Declaration of competing interest

The authors of this manuscript do not have any potential or perceived conflicts of interest to declare.
